# Label-free fibre optic Raman spectroscopy with bounded simplex-structured matrix factorization for the serial study of serum in amyotrophic lateral sclerosis†

**DOI:** 10.1039/d2an00936f

**Published:** 2022-09-28

**Authors:** James J. P. Alix, Nick S. Verber, Chlöe N. Schooling, Visakan Kadirkamanathan, Martin R. Turner, Andrea Malaspina, John C. C. Day, Pamela J. Shaw

**Affiliations:** Sheffield Institute for Translational Neuroscience, University of Sheffield UK j.alix@sheffield.ac.uk pamela.shaw@sheffield.ac.uk; Neuroscience Institute, University of Sheffield UK; Department of Automatic Control and Systems Engineering, University of Sheffield UK; Nuffield Nuffield Department of Clinical Neurosciences, University of Oxford Oxford UK; Blizard Institute, Queen Mary University of London London UK; Interface Analysis Centre, School of Physics, University of Bristol UK

## Abstract

Amyotrophic lateral sclerosis (ALS) is an incurable neurodegenerative disease in urgent need of disease biomarkers for the assessment of promising therapeutic candidates in clinical trials. Raman spectroscopy is an attractive technique for identifying disease related molecular changes due to its simplicity. Here, we describe a fibre optic fluid cell for undertaking spontaneous Raman spectroscopy studies of human biofluids that is suitable for use away from a standard laboratory setting. Using this system, we examined serum obtained from patients with ALS at their first presentation to our centre (*n* = 66) and 4 months later (*n* = 27). We analysed Raman spectra using bounded simplex-structured matrix factorization (BSSMF), a generalisation of non-negative matrix factorisation which uses the distribution of the original data to limit the factorisation modes (spectral patterns). Biomarkers associated with ALS disease such as measures of symptom severity, respiratory function and inflammatory/immune pathways (C3/C-reactive protein) correlated with baseline Raman modes. Between visit spectral changes were highly significant (*p* = 0.0002) and were related to protein structure. Comparison of Raman data with established ALS biomarkers as a trial outcome measure demonstrated a reduction in required sample size with BSSMF Raman. Our portable, simple to use fibre optic system allied to BSSMF shows promise in the quantification of disease-related changes in ALS over short timescales.

## Abstract 2

We demonstrate the use of a fibre optic fluid cell to undertake spontaneous Raman spectroscopy. Advanced advanced matrix factorisation is then applied to identify key spectral patterns with potential improvements to clinical trial design.

## Graphical abstract

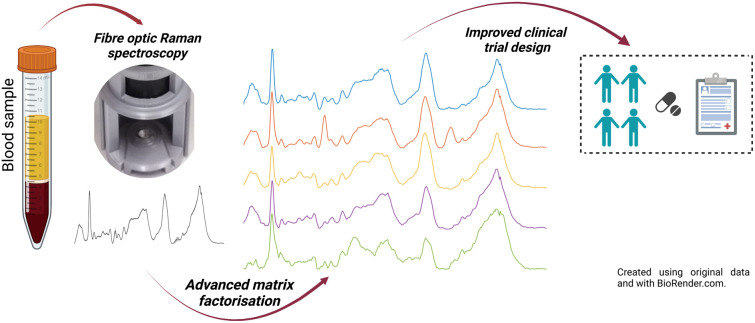

## Introduction

Amyotrophic lateral sclerosis (ALS) is a neurodegenerative condition caused by the progressive loss of motor neurones in the brain and spinal cord. As a result, patients experience weakness of limb and respiratory muscles, as well as of the muscles controlling speech and swallowing. Despite significant progress in understanding the disease, ALS remains incurable with an average survival time of two years from the point of diagnosis.^1^

As a result, many clinical trials are underway which attempt to treat different aspects of the disease. Development of biomarkers of disease that can identify early changes are therefore a priority area of ALS research. Recent imaging,^2^ electrophysiological^3^ and serum^4–6^ biomarker studies have demonstrated early changes in longitudinal measurements (within 3–6 months) with the potential to improve clinical trial design.^7^

Raman spectroscopy is a form of vibrational spectroscopy based upon the inelastic scattering of light. Interest in the application of Raman spectroscopy to neurological disorders is growing due to the simple, label-free nature of the technique.^8,9^ Biofluids are particularly appealing in biomarker research due to the ease of sample acquisition. Thus far, biofluid based ALS studies have typically employed surface enhanced Raman spectroscopy,^10–12^ in which inelastic scattering is potentiated by plasmon excitation in nanoparticles.^13^ Spontaneous Raman spectroscopy, which is more straightforward to implement but generates a far weaker signal, has also been used, albeit less frequently, in studies on ALS^14^ and other neurodegenerative diseases.^15^ While most applications have employed standard laboratory-based microscope formats, fibre optic technologies are gaining momentum due to their potential for use in clinical environments.^16^ Invasive, *in vivo* fibre optic based measurements have been a focus for development but bedside testing of easily obtainable samples, such as blood, would potentially avoid complex laboratory assays and provide rapid results upon which clinicians could act.

Parallel to technological advances, the development of data analysis algorithms is key for the incorporation of Raman spectroscopy into clinical research and, ultimately, clinical practice.^17,18^ With the exception of deep learning methods, most statistical analyses begin with dimension reduction, a process which aims to improve data visualisation and remove redundant information. Non-negative matrix factorisation (NMF) is popular method, used for both dimension reduction and feature extraction within signal processing and biomedical fields.^19^ The technique combines multivariate analysis and linear algebra to decompose the original data into two lower ranking matrices (*i.e.* with fewer dimensions). In the context of Raman spectroscopy, one of these matrices contains the spectral patterns, also termed ‘modes’, for which the non-negative constraint provides a physically realistic output. The other matrix contains the associated coefficients, typically termed ‘weights’, which represent the relative importance of a given pattern to a given sample. Recently, generalisations of NMF, termed simplex structured matrix factorisation, have been proposed which do not impose the non-negative constraint.^20^ A newly developed variant of this approach, termed bounded simple structured matrix factorisation (BSSMF), uses the distribution of the original data to impose bounds on the approximation.^21^ As for other forms of matrix factorisation, two lower rank matrices are produced, however, BSSMF imposes the interval found in the origin data upon the decomposition. Thus, when applied to spectral data, such restrictions should enhance the interpretability of the dominant spectral patterns that are identified.

In this proof-of-concept study, we constructed a portable fibre optic Raman system for the study of biofluids in clinical environments. We studied serum samples collected from ALS patients at two time points and analysed the Raman spectra using BSSMF. We compared Raman data to established clinical measures of disease severity, standard clinical analytes known as promising biomarkers of disease activity (C-reactive protein,^22^ ferritin^23^ and complement^24^) and the leading new serum biomarker for ALS (neurofilament-light, NfL^25,26^). Our data show promise in optimising the biomarker potential of serum Raman studies in ALS.

## Methods

### Participants and clinical assessments

Samples were collected as part of A Multicentre Biomarker Research Strategy in ALS (AMBRoSIA) study, a longitudinal, observational biomarker study. The study was approved by an NHS Research Ethics committee (reference: 16/LO/2136).

66 patients were recruited at their first presentation to the Royal Hallamshire Hospital, Sheffield, UK. After written consent was obtained blood samples were collected and clinical measures of disease completed. Forced vital capacity (FVC), assessment of respiratory function, was measured using a handheld spirometer and a percentage of the patient's predicted value calculated using subject age and height. The ALS Functional Rating Scale-Revised (ALSFRS-R), the established symptom severity score for ALS, was completed. A baseline disease progression rate (DPR) was calculated as (48-ALSFRS-R)/(months from symptom onset at the time of first sample collection). A second data collection visit, comprising repeat clinical assessments and venepuncture, was undertaken 4 months after the first visit in *n* = 27 patients.

### Serum assays

Following venepuncture, samples were separated out for further processing of the different biomarkers. For ferritin, CRP and complement (C3 and C4), samples were sent to the Clinical Chemistry and Clinical Immunology laboratories at Sheffield Teaching Hospitals NHS Foundation Trust. For NfL and Raman studies, serum was separated from blood (centrifuged at 3500 rpm at 4 °C for 10 minutes) and stored in liquid nitrogen. For NfL, these were thawed on ice and quantified using the Mesoscale Discovery (MSD) R-PLEX electrochemiluminescent (ECL)-ELISA platform, as per the manufacturer's instructions.

### Raman spectroscopy

**Fig. 1:**
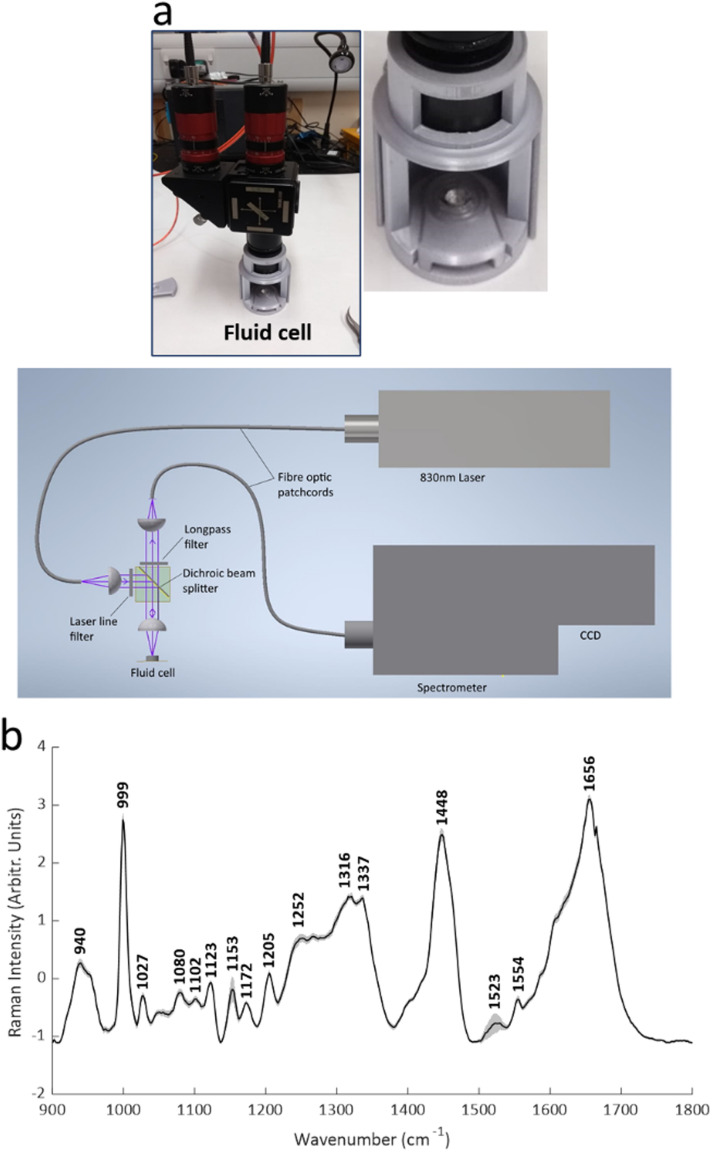
The portable fibre optic Raman system. (a) The 3D printed fluid cell and schematic of the system. (b) Mean (± standard deviation) spectra (all samples).

Raman spectra were obtained from the samples of all patients using a custom made, fibre optic coupled, liquid measurement cell (Fig. 1; Clifton Photonics Ltd, Bristol, UK). This cell focusses laser excitation into a 40 μl disposable aluminium sample container with an objective lens of focal length 25 mm and collects scattered light in reflection mode. The internal optics provide filtering for the rejection of elastically scattered light and clean-up of the incident laser beam. The collection optics have focal length 25 mm and numerical aperture of 0.22. Optical fibres coupled the cell to an 830 nm diode laser (Process Instruments, Salt Lake City, USA) and a Raman Explorer spectrometer (Headwall Photonics Inc. Bolton, Massachusetts, USA). The spectrometer was used in conjunction with an Andor iDus 420 CCD and Andor Solis software (Andor Technology ltd, Belfast UK) for data acquisition.

Acquisition of Raman spectra was undertaken in a windowless hospital clinic room. Serum samples were thawed on ice and 25 μl pipetted into the aluminium sample holder. A laser power of 60 mW measured at the sample was used. PTFE spectra were collected at the start of each recording session for wavenumber calibration. In addition, a background signal was collected with an empty aluminium pan *in situ* within the recording chamber. For each serum sample, an 8 seconds exposure was used, and 20 spectra were recorded (total recording time therefore 160 seconds). The individual spectra were then averaged prior to analysis. Replicates were taken from 20 samples chosen at random; for these, a second 25 μl of serum was taken from the main aliquot and the spectral collection process noted above repeated.

### Analysis

Spectral analyses were undertaken using MATLAB (Version R2021b, The MathWorks, Inc., Natick, MA, USA). Analyses were undertaken across all spectra (*i.e.* visit 1 and visit 2), with separation of the two visits undertaken *post-hoc*. Spectra were first windowed between 900 and 1800 cm^−1^. At <900 cm^−1^ the spectra were dominated by silica-related artefact from the fibre optics. At >1800 cm^−1^ the spectra consisted of non-biological noise. Windowed spectra were interpolated to integer wavenumber spacings, followed by background subtraction using the rubber band algorithm^27^ and standard normal variate (SNV) normalisation. Peaks were identified using the findpeaks MATLAB function.

BSSMF^21^ was also applied across all spectra *i.e.* visit 1 and visit 2 were analysed as one dataset. The data are built up as an *n* × *m* matrix, ***X***, where *n* is the 900 wavenumbers and *m* is the number of spectra (herein, 66 first visit samples plus 27 seconds visit samples equals a total of 93 spectra). For a rank *r* factorisation, (where the rank, *r*, represents the number of spectral patterns output from the factorisation) BSSMF approximates ***X*** as the product of two low rank matrices: ***X*** ≈ ***WH***, where ***W*** has *n* rows and *r* columns, and ***H*** has *r* rows and *m* columns. The unique characteristic of the BSSMF method is that entries in each column of ***W*** are bounded to belong in the interval of the observed dataset ***X***. Thus, the data in the matrix ***W*** represent the dominant spectral patterns in the dataset and are termed ‘modes’. By bounding these modes into the interval of the observed data range we identify spectra which are physically realistic to observe. The columns of ***H*** belong to the unit simplex; these are the weightings and represent the relative importance of each spectral pattern (*i.e.* mode) to each sample. To select the rank of the decomposition, *r*, that is, the number of spectral patterns to be found, the root mean square residual (***R***) between replicate data of 20 samples was calculated:
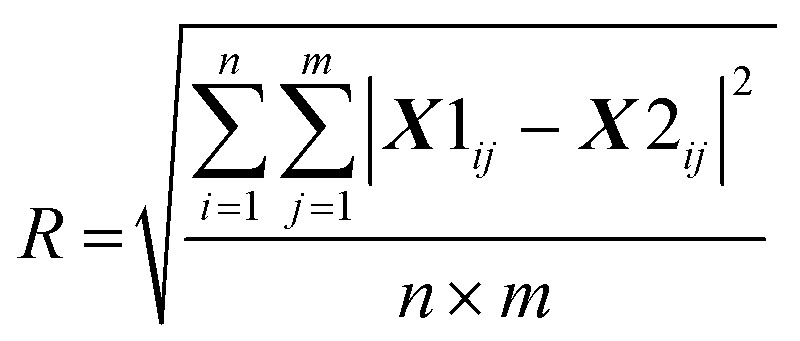
where ***X*1** is the first run of the replicates and ***X*2** is the repeat set (see ESI Fig. S1† for a subtraction spectrum of the technical replicates). For the matrix reconstruction, the root mean square residual (***D***) between the dataset (***X***) and the approximation (***WH***) was determined:
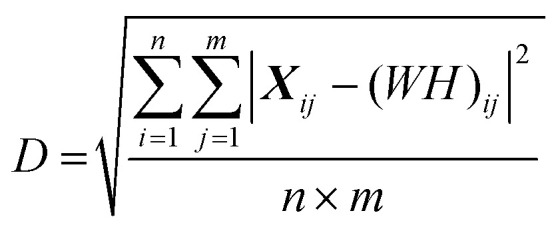


The rank was chosen when the decomposition residual became smaller than the deviation between replicates (*i.e.* when ***D*** < ***R***). This was satisfied for a rank of 5; thus, 5 dominant spectral modes were found (M1–M5). All subsequent analyses were applied to the weighting matrix, ***H***. These included Pearson correlations with the different markers of disease (NfL, CRP, ferritin and complement, FVC, ALSFRS-R, DPR) which were undertaken using visit 1 data. For assessment of visit 1 *vs.* visit 2 repeated measures one-way analysis of variance with a false discovery rate correction (*Q* = 0.05) was performed using GraphPad Prism (Version 9).

**Fig. 2:**
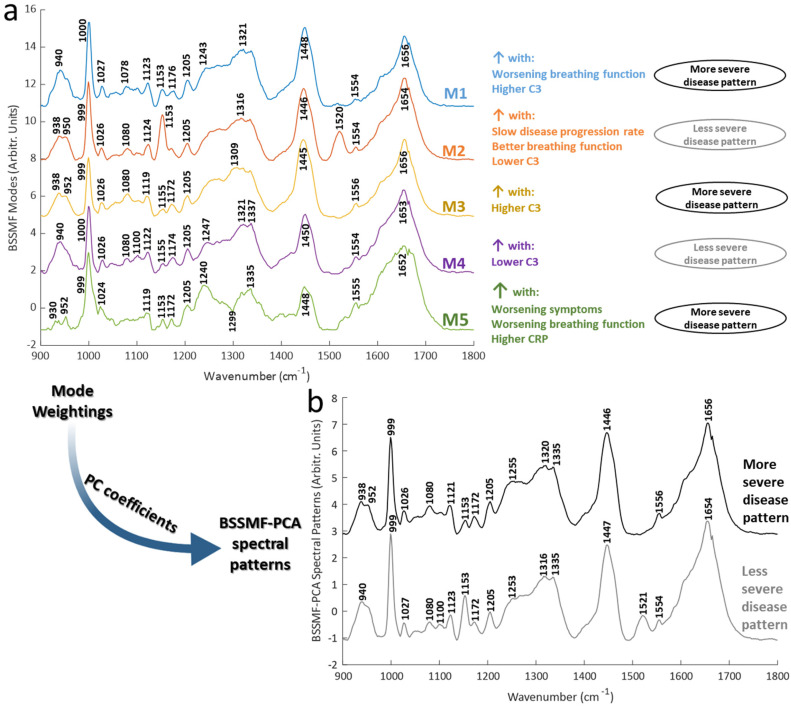
Spectral patterns arising from the BSSMF and their linear combinations. (a) Modes from the BSSMF factorisation of all data. Visit 1 outputs were then correlated with visit 1 clinical/biomarker data to established associations with disease severity (n=66 samples from 66 patients). (b) Modes were combined with PCA and then the relevant PC1 coefficients used to derive spectral patterns associated with worsening disease (modes 1, 3, 5) and less severe disease (modes 2, 4).

In addition, principal component analysis (PCA) was applied to the 5 spectral weightings of the entire dataset, and the direction of maximum variance (PC1) was calculated (BSSMF-PCA). Considering the *j*^th^ sample, the BSSMF-PCA value is given by 
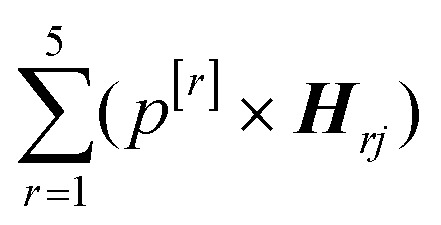
, where *p*^[*r*]^ is the PC1 coefficient for the *r*^th^ dimension. For identification of patterns associated with more/less severe disease the five modes were split into two subgroups, depending upon whether the respective coefficient, *p*^[*r*]^, was positive or negative. If a mode has a positive coefficient, then as its weighting increases, a relative increase in the BSSMF-PCA value is observed. By contrast, increasing weight for the modes with negative coefficients results in a relative decrease in BSSMF-PCA. The PC coefficients were then used to derive a linear combination of the modes for the two subgroups (more/less severe disease). To do this, each BSSMF mode in the subgroup was multiplied by the absolute value of the respective PC1 coefficient, |*p*^[*r*]^|. Hence, by assessing how the BSSMF-PCA value correlates with disease it is possible to assign labels of more severe and less severe disease to these two combined spectral patterns (as shown in Fig. 2b). For the visit 1 *vs.* visit 2 analysis, the paired data (mode weights and BSSMF-PCA scores) were identified for *post hoc* analysis and the same PC coefficient procedure applied.

The sample size for the number of patients required in a hypothetical clinical trial was calculated at 5% significance and 80% power:^28^
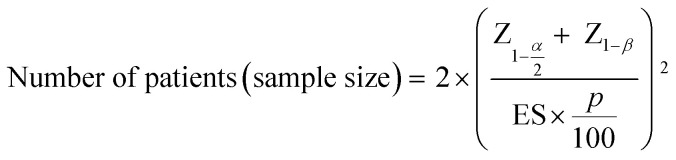
where *Z* is the standard normal distribution for the respective significance level (*α* = 0.05) and power (1 − *β* = 80%), *p* is the treatment effect percentage and ES is the effect size of the biomarker, calculated by assessing the mean change over time (*μ*) and the standard deviation of the change (*σ*):
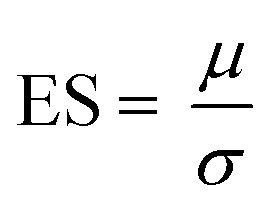


## Results and discussion

### Baseline data

Patient characteristics are shown in Table 1. Raman spectra were obtained and Raman spectra demonstrated features similar to those seen in other blood biofluid studies,^14,29^ with prominent peaks relating to phenylalanine (999 cm^−1^), the amide III region (between 1205 cm^−1^ and 1340 cm^−1^), the CH_2_ deformation of lipids/proteins (1448 cm^−1^) and amide I region (1650–1660 cm^−1^) (Fig. 1). Tentative assignments were taken from the literature (see ESI Table S1† for references).

**Table 1:** Clinical details of the ALS patients

Mean age (s.d.)	62 years (12)
Gender (*n*, %)	Male: 37 (56%)
	Female: 29 (44%)
Site of disease onset (*n*, %)	Limb: 55 (84%)
	Bulbar: 8 (12%)
	Respiratory: 1 (2%)
	Cognitive: 1 (2%)
Mean disease duration (s.d.)	22 months (21.7)
Mean ALSFRS-R score (range)	37 (17–48)
Mean DPR (range)	0.8 (0–6)
Mean % predicted FVC (range)	86 (12–141)

BSSMF was undertaken and the five modes demonstrated similar prominent peaks to the raw spectra (Fig. 2a). Shifts were evident for certain peaks. These included phenylalanine peaks (999/1000 and 1024–1027 cm^−1^), the CH_2_ deformation of lipids/proteins (1446–1450 cm^−1^) and α-helical protein content (938–942 cm^−1^). There were also differences in the prominence, or even presence/absence of other peaks, including 1078–1080 cm^−1^ (present in modes M1–M4), 1153 cm^−1^ (present in M1, M2, M5), 1240 cm^−1^ (M5), 1520 cm^−1^ (prominent in M2). See ESI Table S1† for further mode peak assignments.

Correlations between spectra obtained from samples taken at the baseline visit (*i.e.* visit 1, *n* = 66) and clinical/biochemical characteristics were explored using mode weights. These demonstrated that some modes were associated with more severe disease (M1, M3, M5), and some with less severe disease (M2, M4; Fig. 2a; see ESI Table S2† for full correlation statistics). Particularly prominent correlations were seen for FVC and the inflammatory/immune proteins C-reactive protein (CRP) and C3. FVC is an established respiratory assessment used in ALS. CRP is an acute phase protein which acts as an activator of the complement system, a key component of the innate immune system. Both are increased in ALS and associated with a more severe form of disease.^22,30–34^ In keeping with these prior reports, in our analyses modes associated with increasing CRP/C3 were also associated with worsening disease.

The combined BSSMF-PCA metric was also found to be associated with more severe disease (see ESI Table S2† for correlations and ESI Table S3† for PC coefficients). Thus, more/less severe disease spectral patterns were obtained (Fig. 2b). By using multiple measures of disease this approach provides a more comprehensive means of findings spectral features associated with worsening disease. These patterns demonstrated peak differences related to protein structure (938–952, 1253/55, 1316/1320, 1654/6 cm^−1^) and lipids (1100 cm^−1^). Peak shifts such as these have been described in other diseases, such as malignancy^35,36^ and necrosis.^37^ The understanding of the exact mechanisms driving such shifts is incomplete but may involve altered inter-molecular interactions.^38^ Thus, changes in chemical bond length or symmetry, perhaps related to structural alterations of proteins, may drive such changes.

**Fig. 3:**
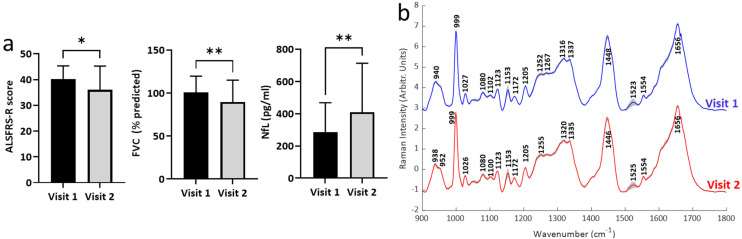
Change in clinical and biochemical measures of disease. (a) Changes in key clinical and biochemical measures of disease (*n* = 27; see ESI fig. S2† for data from other measures). (b) Mean (± standard deviation) for Raman spectra obtained from patients attending both visit 1 and visit 2 (*n* = 27). *p < 0.05, **p < 0.01.

While environmental factors such as sample temperature could also contribute, we noted that other peaks demonstrated alterations in shape and intensity, or were absent in some modes (*e.g.* 1335, 1520 cm^−1^). Instrument calibration was performed prior to each recording session and average spectra from each visit manifested several prominent common peaks (*e.g.* 999, 1080, 1123, 1153, 1205, 1554, 1656 cm^−1^; Fig. 3). These observations would suggest that systematic differences in recording conditions were not the dominant reason for peak differences found in the matrix decomposition.

Interestingly, peak alterations indicative of changes in protein structure have also been identified in Raman serum studies of other neurodegenerative conditions.^15^ These disorders, which include ALS, are increasingly recognised as proteinopathies, in which misfolded proteins play a key driving role in disease initiation and spread.^39^ For example, analysis of protein aggregates within blood from ALS patients demonstrated the presence of a large number of proteins involved in the proteasome, the clearance system for defective proteins, illustrating that the effects of abnormal proteins can spread across the blood–brain barrier.^40^ Assessing whether Raman spectroscopy could provide a simple point of care assay on such aggregates would be a useful future work. In addition, peaks associated with carotenoids were more prominent in the less severe disease pattern (1153, 1521 cm^−1^). Carotenoids exhibit free radial scavenging and interact with the Nrf2 signalling pathway, both of which have been implicated in ALS.^41,42^ Interestingly, increased carotenoid intake has been shown to correlate with less severe disease,^43^ although the relationship between intake and disease is likely to be complex.^44^

### Change over time: visit 1 *vs.* visit 2

**Fig. 4:**
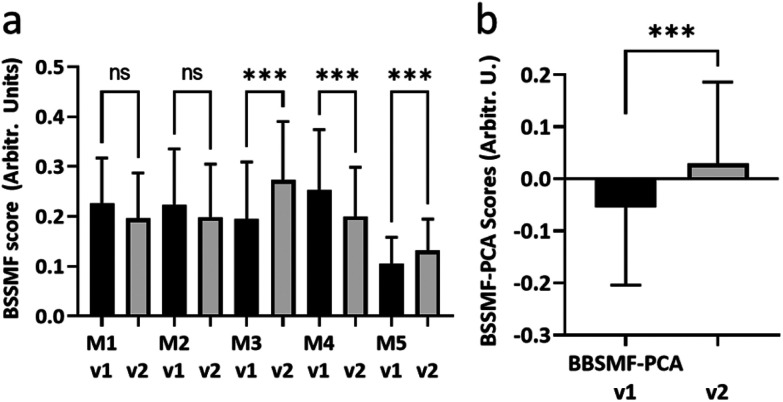
Longitudinal BSSMF mode and BSSMF-PCA changes. (a) Changes in individual BSSMF mode weights. (b) Change in BSSMF-PCA scores from visit 1 and visit 2. ns – not significant, ***p < 0.001.

Second visit assessments were performed 4 months after the first visit in *n* = 27 patients. Established clinical (ALSFRS-R, FVC) and biochemical biomarkers (NfL) demonstrated significant change between the two visits, indicating disease progression in these patients (Fig. 3a; see ESI Fig. S2† for remaining biomarkers). The weightings of modes M3, M4 and M5 from the BSSMF also demonstrated significant changes (Fig. 4a). The BSSMF-PCA scores increased over time (moving from negative to positive; Fig. 4b), which represents a shift from the less severe to the more severe spectral pattern in the earlier analysis. Significant differences were observed in the visit 1/visit 2 BSSMF-PCA scores (*p* = 0.0002; Fig. 4b).

Several reports utilising more traditional techniques for monitoring ALS (such electrophysiological and imaging studies) have demonstrated significant changes in disease state within 4 months.^2,3^ The most promising serum biomarker at present is NfL, a structural protein found in axons which is released when axons degenerate. Recent multi-centre work from the AMBRoSIA study group found that levels increased over time and modelling its inclusion as a trial outcome measure reduced sample size requirements.^26^

To investigate the potential of our Raman paradigm in a longitudinal setting, a hypothetical clinical trial was constructed and sample sizes for the number of patients required (not the volume of serum) were calculated (Table 2). In comparison to established ALS biomarkers (ALSFRS-R, FVC, NfL), Raman data from modes M3-5 and the linear combination of all modes (BSSMF-PCA) required smaller sample sizes.

**Table 2:** Hypothetical clinical trial sample sizes

	Effect size	Sample size: 50% treatment effect	Sample size: 20% treatment effect
**Raman**			
M1	−0.35	529	3310
M2	−0.26	941	5883
M3	0.89	80	502
M4	−0.85	87	547
M5	0.84	89	557
BSSMF-PCA	0.85	87	547
ALSFRS-R	−0.44	323	2024
FVC	−0.78	102	642
Neurofilament-light	0.7	128	798

Fibre optic Raman systems have been investigated for *in vivo* applications, for example, for deployment during surgery and endoscopy.^16^ Serum studies have largely employed either standard microscope formats (*e.g.*^45^), but smaller portable systems are being investigated (*e.g.*^46^). More recently, optofluidic systems, which combine microfluidics to separate blood components with Raman, have been developed.^47,48^ Whether using a portable system such as ours, or a miniature optofluidic design, being able to take Raman to the clinic offers the possibility for real-time molecular fingerprinting at the point of care. Such information could help guide clinical decision making in the precision medicine era. This might benefit a range of diseases, as well as a variety of care settings, for example, emergency care, surgery and outpatient clinics. In addition to immediate patient benefit, reduced decision-making times can also have potential knock-on effects in cost benefit analyses. Developing an evidence base for point of care Raman spectroscopy through high quality, multi-centre studies with parallel health economic assessments, will be a priority for the field as it continues to develop.

The complexity of Raman spectra and the subtle molecular differences found in disease necessitates multivariate analysis, often beginning with dimensionality reduction. The typical approach (PCA) can distort the spectral profile and present physically unrealistic results. Non-negative matrix factorisation can overcome some of these issues and provide a quantitative measure of the importance of different spectral patterns to disease.^49,50^ The bounded simplex structured matrix factorisation used here does not impose the non-negative constraint; instead the factorisation matrix ***W*** is bound within an interval defined by the original data.^21^ Thus, the data within ***W*** can be interpreted in the same way as the original data (***X***). Bounded component analysis approaches are similar in their approach, although more difficult to interpret.^51^ In our analyses we also combined modes to gain a more complete picture of the biochemical changes occurring over time. Analysing Raman data in this fashion could facilitate clinical trials, with reduced sample size numbers reducing trial costs. This, in turn, can help utilise resources more efficiently, meaning more studies could be funded in the search for new treatments. Furthermore, the integration of advanced analysis algorithms with portable fibre optic technology, or indeed other platforms, can aid the development of biomedical Raman spectroscopy as a point of care technology.

## Conclusions

Herein, we have presented a novel, portable fibre optic system for the study of human biofluids. As the development of biomarkers for monitoring ALS is an area of intense investigation, we tested the system on serum from ALS patients collected at a 4-month interval. We analysed data using a matrix decomposition technique with enhanced physical interpretation constraints. Key spectral features appeared to relate to protein structure. Further assessment of serum samples over longer time periods could provide additional insights into the complex biochemical changes occurring the serum of patients with ALS and facilitate more efficient clinical trial design.

## Author contributions

J. J. P. A.: conceptualisation, formal analysis, investigation, methodology, visualisation, writing – original data. N. S. V.: conceptualisation, investigation, project administration, writing – review & editing; C. S.: formal analysis, writing – review & editing; V. K.: writing – review & editing; M. R. T. – funding acquisition, writing – review & editing; A. M. – funding acquisition, writing – review & editing; J. C. C. D,: conceptualisation, methodology, resources, writing – review & editing; P. J. S.: conceptualisation, funding acquisition, resources, project administration, writing – review & editing.

## Conflicts of interest

J. C. C. D. is the chief executive office of Clifton Photonics Ltd, a company that designs photonic instruments for industrial and research use.

## Supplementary Material

AN-147-D2AN00936F-s001
