# Supplementary material for: Label-free fibre optic Raman spectroscopy with bounded simplex-structured matrix factorization for the serial study of serum in amyotrophic lateral sclerosis
Source: Analyst. 2022 Sep 28;147(22):5113–20. doi: 10.1039/d2an00936f (PMC9639415; doi:10.1039/d2an00936f)
Supplement: AN-147-D2AN00936F-s001 [file AN-147-D2AN00936F-s001.pdf]

Electronic Supplementary Material (ESI) for Analyst.
This journal is © The Royal Society of Chemistry 2022

| Wavenumber/wavenumber region ($cm^{-1}$) | Mode and wavenumber ($cm^{-1}$) | Assignment |
|---|---|---|
| 930-942 | M1, M4: 940; M2, M3: 938; M4: 940; M5: 930 | Protein (proline, α-helix)[1, 2] |
| 950/952 | M2: 950; M3, M5: 952 | C-C stretch: protein (proline, valine, α -helix)[3, 4] |
| 999-1000 | M1, M4: 1000; M2, M3, M5: 999 | Phenylalanine (breathing mode) [5] |
| 1024-1027 | M1: 1027; M2, M3, M4: 1026; M5: 1024 | Phenylalanine[6-9] |
| 1078/80/1100 | M1: 1078; M2, M3, M4: 1080; M4: 1100 | Lipids[4, 10-12] |
| 1119-1124 | M1: 1123; M2: 1124, M3, M5: 1119; M4: 1122 | Proteins, lipids[11, 13] |
| 1153/5 | M1, M2, M5: 1153; M3, M4: 1155 | Carbohydrates/carotenoids[5, 14, 15] |
| 1172-1176 | M1: 1176; M3, M5: 1172; M4: 1174 | Tyrosine[11, 16] |
| 1205 | M1-5 | Tyrosine, phenylalanine[2, 17] |
| 1240-1300 | M1: 1243; M4: 1247, M5: 1299 | Proteins: amide III<br>- 1243 β-sheet; 1266-1300 α-helix[18] |
| 1309-1321 | M1: 1321; M2: 1316; M3: 1309; M4: 1321 | DNA/RNA, protein[19] |
| 1335/7 | M4: 1337; M5: 1335 | $CH_3CH_2$ deformation, proteins/lipids[20] |
| 1445-1450 | M1, M5: 1448; M2: 1446; M3: 1445; M4: 1450 | CH modes ($CH_2$ and $CH_3$ deformations: bending & scissoring) in proteins/lipids[5, 21] |
| 1520-5 | M2:1520; BSSMF-PCA: 1521; visit 1: 1523; visit 2: 1525 | Carotenoids[22, 23] |
| 1554/6 | M1, M2, M4: 1554; M3: 1556; M5: 1555 | Tryptophan, proteins[24, 25] |
| 1652/6 | M1, M3: 1656; M2: 1654; M4: 1653; M5: 1652 | Proteins (amide I)[2, 26, 27] |

**Supplementary table 1. Tentative wavenumber assignments.**

| | M1 | M2 | M3 | M4 | M5 | BSSMF- |
|---|---|---|---|---|---|---|

| | | | | | | PCA |
|---|---|---|---|---|---|---|
| **ALSFRS-R** | *r 0.03*<br>*p 0.8* | *r 0.17*<br>*p 0.17* | *r -0.11*<br>*p 0.37* | *r 0.09*<br>*p 0.45* | ***R -0.29***<br>***p 0.01*** | *r -0.19*<br>*p 0.12* |
| **DPR** | *r -0.03*<br>*p 0.8* | ***r -0.31***<br>***p 0.01*** | *r 0.12*<br>*p 0.3* | *r 0.09*<br>*p 0.48* | *r 0.21*<br>*p 0.09* | *r 0.2*<br>*p 0.1* |
| **FVC** | ***r -0.28***<br>***p 0.02*** | ***r 0.39***<br>***p 0.001*** | *r -0.12*<br>*p 0.34* | *r 0.19*<br>*p 0.12* | ***r -0.37***<br>***p 0.002*** | ***r -0.4***<br>***p 0.001*** |
| **NfL** | *r -0.1*<br>*p 0.39* | *r 0.04*<br>*p 0.7* | *r -0.003*<br>*p 0.9* | *r 0.08*<br>*p 0.5* | *r -0.04*<br>*p 0.7* | *r -0.07*<br>*0.6* |
| **Ferritin** | *r 0.12*<br>*p 0.3* | *r -0.04*<br>*p 0.8* | *r 0.03*<br>*p 0.8* | *r -0.15*<br>*p 0.2* | *r 0.08*<br>*p 0.5* | *r 0.1*<br>*p 0.4* |
| **CRP** | *r -0.03*<br>*p 0.8* | *r -0.2*<br>*p 0.11* | *r 0.2*<br>*p 0.11* | *r -0.19*<br>*p 0.13* | ***r 0.37***<br>***p 0.003*** | ***r 0.29***<br>***p 0.02*** |
| **C3** | ***r 0.28***<br>***p 0.02*** | ***r -0.41***<br>***p 0.0007*** | ***r 0.39***<br>***p 0.001*** | ***r -0.32***<br>***p 0.008*** | *r 0.18*<br>*p 0.14* | ***r 0.55***<br>***<0.0001*** |
| **C4** | *r -0.05*<br>*p 0.68* | *r -0.17*<br>*p 0.16* | *r 0.2*<br>*p 0.1* | *r -0.02*<br>*p 0.86* | *r 0.06*<br>*p 0.6* | *r 0.2*<br>*p 0.11* |

**Supplementary table 2.** Correlation of baseline BBSMF mode weights and clinical/biochemical markers of disease.

| **Mode** | **PC1 coefficients** |
|---|---|
| M1 | 0.1930 |
| M2 | -0.6114 |
| M3 | 0.6256 |
| M4 | -0.4003 |
| M5 | 0.1931 |

**Supplementary table 3. PC1 coefficients.**

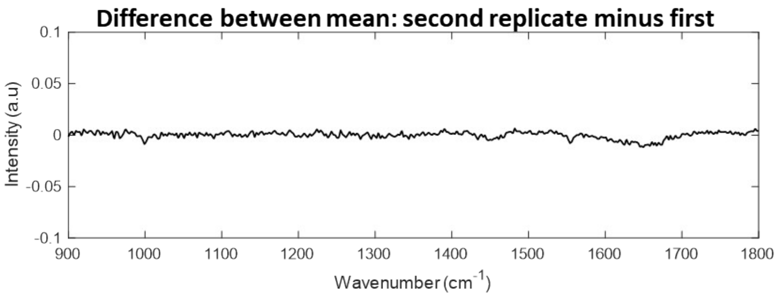

**Supplementary figure 1.** Difference between the mean plot for the technical replicates (2nd replicate minus the 1st).

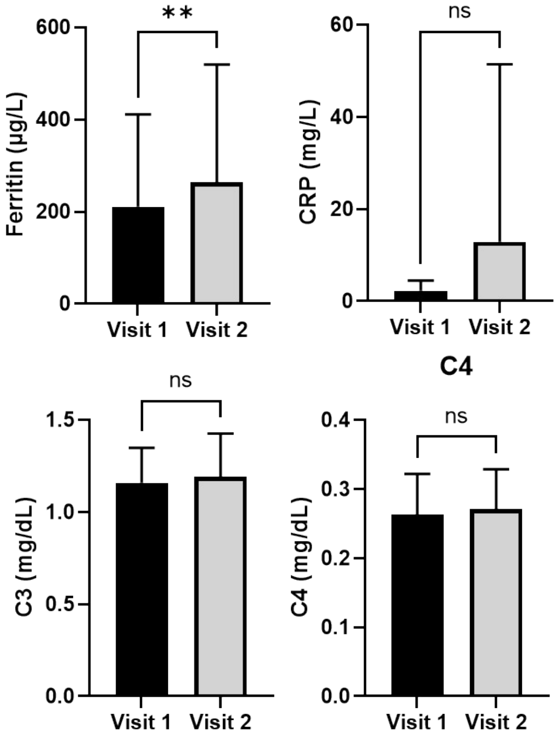


**Supplementary figure 2.** Changes between the two study visits for additional biochemical markers of disease.

**References**


1. F. Bonnier and H. J. Byrne, *Analyst*, 2012, **137**, 322-332.
2. N. Stone, C. Kendall, J. Smith, P. Crow and H. Barr, *Faraday Discuss*, 2004, **126**, 141-157; discussion 169-183.
3. A. Plante, F. Dallaire, A.-A. Grosset, T. Nguyen, M. Birlea, J. Wong, F. Daoust, N. Roy, A. Kougioumoutzakis, F. Azzi, K. Aubertin, S. Kadoury, M. Latour, R. Albadine, S. Prendeville, P. Boutros, M. Fraser, R. Bristow, T. Van Der Kwast, M. Orain, H. Brisson, N. Benzerdjeb, H. Hovington, A. Bergeron, Y. Fradet, B. Têtu, F. Saad, D. Trudel and F. Leblond, *Journal of Biomedical Optics*, 2021, **26**, 116501.
4. M. Gniadecka, H. C. Wulf, N. Nymark Mortensen, O. Faurskov Nielsen and D. H. Christensen, *Journal of Raman Spectroscopy*, 1997, **28**, 125-129.
5. N. Stone, C. Kendall, N. Shepherd, P. Crow and H. Barr, *Journal of Raman Spectroscopy*, 2002, **33**, 564-573.
6. L. M. Almehmadi, S. M. Curley, N. A. Tokranova, S. A. Tenenbaum and I. K. Lednev, *Scientific Reports*, 2019, **9**, 12356.
7. A. Stefancu, V. Moisoiu, C. Bocsa, Z. Bálint, D.-T. Cosma, I. A. Veresiu, V. Chiş, N. Leopold and F. Elec, *Analyst*, 2018, **143**, 5372-5379.
8. Y. Yang, Y. Peng, C. Lin, L. Long, J. Hu, J. He, H. Zeng, Z. Huang, Z.-Y. Li, M. Tanemura, J. Shi, J. R. Lombardi and X. Luo, *Nano-Micro Letters*, 2021, **13**, 109.
9. R. A. Karaballi, S. Merchant, S. R. Power and C. L. Brosseau, *Physical Chemistry Chemical Physics*, 2018, **20**, 4513-4526.
10. X. Ren, K. Lin, C.-M. Hsieh, L. Liu, X. Ge and Q. Liu, *Biomed. Opt. Express*, 2022, **13**, 344-357.
11. Z. Huang, A. McWilliams, H. Lui, D. I. McLean, S. Lam and H. Zeng, *Int J Cancer*, 2003, **107**, 1047-1052.
12. V. O. Baron, M. Chen, B. Hammarstrom, R. J. H. Hammond, P. Glynne-Jones, S. H. Gillespie and K. Dholakia, *Communications Biology*, 2020, **3**, 236.
13. N. Huang, M. Short, J. Zhao, H. Wang, H. Lui, M. Korbelik and H. Zeng, *Opt Express*, 2011, **19**, 22892-22909.
14. D. K. R. Medipally, D. Cullen, V. Untereiner, G. D. Sockalingum, A. Maguire, T. N. Q. Nguyen, J. Bryant, E. Noone, S. Bradshaw, M. Finn, M. Dunne, A. M. Shannon, J. Armstrong, A. D. Meade and F. M. Lyng, *Therapeutic Advances in Medical Oncology*, 2020, **12**, 1758835920918499.
15. B. Mooij, B. J. A. Mooij, I. H. M. van Stokkum, G. R. Davies and F. Ariese, *Journal of Optics*, 2022.
16. A. Daniel, A. Prakasarao, B. David, L. Joseph, C. Murali Krishna, K. D and S. Ganesan, *Journal of Raman Spectroscopy*, 2014, **45**, 541-549.
17. J. De Gelder, K. De Gussem, P. Vandenabeele and L. Moens, *Journal of Raman Spectroscopy*, 2007, **38**, 1133-1147.
18. C. J. Kirkby, J. Gala de Pablo, E. Tinkler-Hundal, H. M. Wood, S. D. Evans and N. P. West, *Analyst*, 2021, **146**, 581-589.
19. A. C. S. Talari, Z. Movasaghi, S. Rehman and I. u. Rehman, *Applied Spectroscopy Reviews*, 2015, **50**, 46-111.
20. A. J. Ruiz-Chica, M. A. Medina, F. Sánchez-Jiménez and F. J. Ramírez, *Journal of Raman Spectroscopy*, 2004, **35**, 93-100.
21. L. Silveira, Jr., F. L. Silveira, B. Bodanese, R. A. Zangaro and M. T. Pacheco, *J Biomed Opt*, 2012, **17**, 077003.
22. F. E. R. Woods, S. Chandler, N. Sikora, R. Harford, A. Souriti, H. Gray, H. Wilkes, C. Lloyd-Bennett, D. A. Harris and P. R. Dunstan, *Clinical Spectroscopy*, 2022, **4**, 100020.
23. J. L. Gonzalez-Solis, J. C. Martinez-Espinosa, L. A. Torres-Gonzalez, A. Aguilar-Lemarroy, L. F. Jave-Suarez and P. Palomares-Anda, *Lasers Med Sci*, 2014, **29**, 979-985.

24. R. Gautam, S. Vanga, A. Madan, N. Gayathri, U. Nongthomba and S. Umapathy, *Anal Chem*, 2015, **87**, 2187-2194.
25. E. B. Carew, I. M. Asher and H. E. Stanley, *Science*, 1975, **188**, 933-936.
26. A. Mahadevan-Jansen and R. R. Richards-Kortum, *J Biomed Opt*, 1996, **1**, 31-70.
27. A. Mahadevan-Jansen, M. F. Mitchell, N. Ramanujam, A. Malpica, S. Thomsen, U. Utzinger and R. Richards-Kortum, *Photochem Photobiol*, 1998, **68**, 123-132.
